# Distension evoked mucosal secretion in human and porcine colon *in vitro*

**DOI:** 10.1371/journal.pone.0282732

**Published:** 2023-04-13

**Authors:** Kristin Elfers, Stefanie Schäuffele, Susanne Hoppe, Klaus Michel, Florian Zeller, Ihsan Ekin Demir, Michael Schemann, Gemma Mazzuoli-Weber

**Affiliations:** 1 Institute for Physiology and Cell Biology, University of Veterinary Medicine Hannover, Hannover, Germany; 2 Chair of Human Biology, Technical University of Munich, Freising, Germany; 3 Academic Hospital Freising, Freising, Germany; 4 University Hospital Rechts der Isar, Technical University of Munich, München, Germany; 5 Center for Systems Neuroscience (ZSN), Hannover, Germany; Cinvestav-IPN, MEXICO

## Abstract

It was suggested that intestinal mucosal secretion is enhanced during muscle relaxation and contraction. Mechanisms of mechanically induced secretion have been studied in rodent species. We used voltage clamp Ussing technique to investigate, in human and porcine colonic tissue, secretion evoked by serosal (P_ser_) or mucosal (P_muc_) pressure application (2–60 mmHg) to induce distension into the mucosal or serosal compartment, respectively. In both species, P_ser_ or P_muc_ caused secretion due to Cl^-^ and, in human colon, also HCO_3_^-^ fluxes. In the human colon, responses were larger in proximal than distal regions. In porcine colon, P_muc_ evoked larger responses than P_ser_ whereas the opposite was the case in human colon. In both species, piroxicam revealed a strong prostaglandin (PG) dependent component. P_ser_ and P_muc_ induced secretion was tetrodotoxin (TTX) sensitive in porcine colon. In human colon, a TTX sensitive component was only revealed after piroxicam. However, synaptic blockade by ω-conotoxin GVIA reduced the response to mechanical stimuli. Secretion was induced by tensile rather than compressive forces as preventing distension by a filter inhibited the secretion. In conclusion, in both species, distension induced secretion was predominantly mediated by PGs and a rather small nerve dependent response involving mechanosensitive somata and synapses.

## Introduction

The enteric nervous system (ENS), with its myenteric plexus (MP), located between the two muscle layers and its submucosal plexus (SMP), located beneath the mucosa, is capable of generating local reflex circuits regulating gastrointestinal motor and secretory functions independently from central input [1–3]. In rodent models, nerve evoked secretory responses of the intestinal epithelium are almost entirely due to increase in serosal to mucosal Cl^-^ flux, which is additionally modulated by endocrine, paracrine, autocrine and immunological factors [for review see 4,5]. In human intestine, there is an additional contribution of HCO_3_^-^ to nerve and pharmacologically evoked anion secretion [6].

Mechanical deformation, such as mucosal stroking or distention of the intestinal wall, evoked secretion in different intestinal segments of various species [7–11]. Since mechanical stimuli simultaneously initiate the peristaltic reflex and epithelial secretion, it is assumed that this coordinated response facilitates propulsion of intestinal contents through the intestinal lumen and avoids stasis [8,10]. The first observation of an association between muscle movements, measured by intraluminal pressure changes and net epithelial ion transport, recorded by transmural potential differences (PD), were from human and canine jejunum *in vivo* [12]. This seminal study showed that contractions were closely associated by increased PD which peaked 40 sec after the pressure peak. Similarly, distension by intraluminal bolus injection evoked increased PD which peaked 60 sec after the maximum distension was reached. *In vivo* studies in the ferret jejunum demonstrated an association between PD and bursts of muscle contractions which required input from the ENS [13]. A later study in rat jejunum showed that the SMP rather than the MP is crucial for temporal correlation between muscle activity and epithelial secretion [14].

Over time, studies in isolated gut segments complemented the above in vivo findings in order to provide some mechanistic insights into secretory activity triggered by defined mechanical stimuli. Such studies were performed in mice, rats and guinea pigs to conclude that distension or mucosal deformation evoked anion secretion involving nerves and a plethora of neuro humoral mediators [4,8–10,15,16]. The involvement of prostaglandins (PGs) have been found in the rat colon [9,17], but seemed to be less important in the guinea pig colon [10]. Another species-related difference was the strong contribution of acetylcholine (ACh) to distension evoked secretion in the guinea pig, whereas the cholinergic component was very weak in the rat colon. Besides species-specific neural components, secretory responses were also dependent on how mechanical stimulation was performed. While mucosal stroking does initially involve serotonin (5-HT) release from enterochromaffin cells [18–20], this mechanism was not operative during distension in the guinea pig colon [10,16].

Unlike for rodents, distension evoked secretion has not been studied in the human colon. In the field of gastrointestinal physiology, the pig became more and more popular as a model for the human. Although several reviews propagated this suggestion, original studies looking at neural reflexes in the porcine intestine are very scarce [for review see 21,22]. The few comparative studies would rather suggest important differences between porcine and human intestinal secretion. Thus, nerve evoked secretion is mainly HCO_3_^-^ and to a lesser extent, Cl^-^ driven in the human colon [6], whereas secretion of Cl^-^ is dominant in the pig colon [23]. The intrinsic nerve pathways involved in anion secretion are partially cholinergic in porcine colon [24]. However, in the human colon the neurotransmitter VIP plays a major role [6]. Nevertheless, ACh via activation of muscarinic receptors induced secretion in the human as well as in the porcine colon [6,25–27]. Despite these functional differences, we recently found that the major features of mechanosensitive neurons in the porcine and human submucous plexus were comparable [28].

The aim of the present study was to provide novel insights into distension evoked epithelial secretion in the human colon *in vitro* using the Ussing chamber voltage clamp technique and to address with selected experiments similarities and differences between human and pig. We found that distension from the mucosal or serosal side, which mimicked relaxation and contractions of the colonic wall, respectively, evoked secretion in both species. Findings in human samples showed that a TTX sensitive component was only revealed after blockade of PG synthesis. Interestingly, ω-conotoxin attenuated the response to distension in the human colon suggesting involvement of mechanosensitive synapses. In contrast, the TTX sensitive component of distension induced secretion was readily revealed in the pig colon suggesting that distension induced somal activation is more obvious in the porcine colon. However, considering both, TTX and ω-conotoxin sensitive components of the secretory response, the overall proportion of distension induced secretion mediated by nerve activity was less than 30% in both, human and pig, and was, therefore, relatively small. In both, porcine and human colon, PGs played a major role in distension evoked secretion. While the mechanically induced secretion was primarily carried by Cl^-^ in the pig colon, HCO_3_^-^ and Cl^-^ were equally important in the human colon. In both species, the secretory response was dependent on tissue distension rather than compression.

## Material and methods

### Tissue samples

#### Porcine tissue

To reduce number of animal experiments according to the 3Rs concept of reduction, replacement and refinement implemented in the Directive 2010/63/EU, we used colonic tissue from hybrid pigs (female line German Large White × German Landrace; male line Pietrain) of both sexes (24 weeks old) which were bred and fattened on local farms and finally slaughtered for food production at a local slaughterhouse (Leinefleisch GmbH Laatzen, Hannover, Germany). Samples of the transverse colon (n = 80) were taken approximately 10 cm distal from the last spiral gyri centrifugales of the spiral colon (see S1 Fig), directly after removal of the intestinal tract from the carcass. We used only the transverse colon since the porcine proximal colon has a unique anatomical structure not including a direct correlate of the human proximal colon. Preparation of the porcine descending colon as it would have been necessary for Ussing chamber experiments, was not successful. Samples were immediately placed into ice cold, carbogen aerated (95% CO_2_, 5% O_2_; pH 7.40) Krebs solution containing (in mM) 117 NaCl, 4.7 KCl, 1.2 MgCl_2_, 1.2 NaH_2_PO_4_, 25 NaHCO_3_, 2.5 CaCl_2_ and 11 glucose and transported to the laboratory. Mucosa/submucosa preparations containing the inner SMP were obtained by removing the serosa, muscle layers and the outer SMP embedded into fat tissue.

#### Human tissue

In total, 540 human tissue samples of the large intestine were obtained from 108 patients (60 male, 48 female) undergoing surgery at the Medical Clinics in Freising, Erding and Rechts der Isar (Munich, Germany). Samples were taken from macroscopically unaffected areas and were immediately inspected by a pathologist and transported to the laboratory for dissection of mucosa/submucosa preparations. Buffer solution used for transport and dissection of the samples were the same as described for porcine tissues. All procedures were performed in accordance with the Declaration of Helsinki (Ethical Principles for Medical Research Involving Human Subjects) and were approved by the ethics committee of the Technische Universität München (5242/11). Written informed consent was given by all patients. Table 1 summarizes the intestinal regions studied and the underlying diagnosis for surgical resection.

**Table 1:** Location of human colonic specimens and diseases underlying the surgery.

ascending colon	transverse colon	descending colon	sigma/rectum
colon cancer (16)	colon cancer (13)	colon cancer (10)	colon cancer (25)
diverticulitis (1)	colon polyps (4)	diverticulitis (2)	diverticulitis (23)
Morbus Crohn (1)		unspecified (2)	Morbus Crohn (4)
unspecified (4)			colon polyps (1)
			unspecified (2)

Numbers in brackets indicate the number of patients.

### Ussing chamber experiments

We used the Ussing chamber technique as an established in vitro setup for measuring transport processes across intestinal epithelia (*Ussing HH*, *Zerahn K*. *Active transport of sodium as the source of electric current in the short-circuited isolated frog skin*. *Acta Physiol Scand*. *1951; 23 (2–3)*:*110–27*.) In this system, intestinal tissues are fixed between the two halves of a chamber forming a mucosal and a serosal compartment that are filled with an appropriate buffer solution (see drugs and solutions for composition of buffer solutions used for the current experiments). The movement of ions across the intestinal epithelium produces a potential difference (PD), which is set to 0 mV under the applied short circuit (I_sc_) conditions, using a voltage clamp device (EC-285, Warner Instruments). Under such conditions, recorded changes in I_sc_ indicate net transepithelial ion transfer with positive ΔI_sc_ either representing absorption of cations (such as Na^+^) or secretion of anions such as chloride (Cl^-^). Colonic mucosa-submucosa preparations, were mounted into Ussing chambers (serosal area 1.08 cm^2^) and were allowed to equilibrate for 45 to 60 min. After equilibration, basal I_sc_ was measured and tissue conductance (G_t_) was assessed for evaluation of tissue integrity, with intact epithelia known to exhibit a basal tissue resistance and a basal I_sc_. This was followed by mucosal application of 10 μM Amilorid (A-7410, Sigma-Aldrich, Taufkirchen, Germany) dissolved in dimethytolsulfoxide (DMSO) to prevent Na^+^ driven increases in I_sc_. An electrical field stimulation (EFS; 10 s train pulse with 1 ms single pulse duration at 20 V (human tissue) and 30 V (porcine tissue)) was applied by a constant voltage stimulator (Grass SD9 and SD48; Astro-Med Inc., West Warwick, RI, USA) connected to platinum electrodes in order to evoke a neuronal mediated increase in I_sc_ revealing tissue viability prior to pressure application. Only tissues exhibiting an EFS-induced increase in I_sc_ suggesting active transepithelial ion secretion were used for further experiments. 80% of the human tissue responded to the EFS. Tissue distension was evoked by a 60 s lasting pressure application from the serosal or mucosal side using a self-constructed syringe pump, operated by an Arduino microcontroller, injecting volume into the closed Ussing chamber until the predefined pressure was reached (Michel, K., Schemann, M., 2020. Ussing Chamber Pressure Pump. https://doi.org/10.26275/4QVR-KWZQ). In between two tissue distensions pressure was completely scaled back to reach basal I_sc_ values again. In a first set of experiments, increasing serosal pressures of 10 mmHg (only human tissue), 20 mmHg and 60 mmHg were applied to evaluate pressure strength-dependency of the secretory response and to determine pressure values leading to comparable secretory responses in human and porcine tissues, respectively. Reproducibility of the secretory response induced by application of a 20 mmHg (human) and 60 mmHg (porcine) pressure was validated by distending tissues three times from the serosal side. In paired experimental designs, the following treatments were applied prior to distension: (a) replacement of the normal Krebs solution by Cl^-^ and Cl^-^ and HCO_3_^-^ free buffer solutions, (b) serosal addition of the nerve blocker tetrodotoxin (TTX, 1 μM), (c) serosal addition of the cyclooxygenase (COX) inhibitor piroxicam (10 μM) and in combination with TTX, (d) serosal addition of PGE_2_ (1 μM) with and without prior incubation with TTX, (e) serosal addition of 500 nM ω-conotoxin GVIA, a blocker of N-type voltage-dependent Ca channels, blocking synaptic transmission (only human tissue), (f) serosal application of the choline esterase inhibitor neostigmine (1 μM) and in combination with TTX (only human tissue), and (g) a filter placed on the mucosal side impeding distension during serosal pressure application.

### Drugs, solutions and filter

The apical and basolateral compartments of the Ussing chambers were filled with 10 ml carbogen aerated Krebs solution containing (in mM) 117 NaCl, 4.7 KCl, 1.2 MgCl_2_, 1.2 NaH_2_PO_4_, 20 NaHCO_3_, 2.5 CaCl_2_ and 11 glucose (all from Sigma-Aldrich, Taufkirchen, Germany), maintained at 37°C. For experiments with Cl^-^ free buffer solution, Krebs solution was replaced by a carbogen aerated buffer solution containing (in mM) 58.5 NaSO_4_, 2.4 KSO_4_, 1.2 MgSO_4_, 1.2 NaH_2_PO_4_, 20 NaHCO_3_, 2.5 CaSO_4_, 11 glucose and 85 mannitol. For experiments with Cl^-^ and HCO_3_^-^ free buffer solution, Krebs solution was replaced by an oxygen aerated buffer solution containing (in mM) 58.5 NaSO_4_, 2.4 KSO_4_, 1.2 MgSO_4_, 1.2 NaH_2_PO_4_, 20 HEPES, 2.5 CaSO_4_, 11 glucose and 91 mannitol. Stock solutions of TTX (Carl Roth GmbH and Co. KG, Karlsruhe, Germany), ω-conotoxin GVIA (Alomone Labs, Jerusalem, Israel) dissolved in distilled water, as well as stock solutions of Piroxicam (P-5654, Sigma-Aldrich,) dissolved in chloroform and prostaglandin E_2_ (PGE_2_) (P5640, Sigma-Aldrich) dissolved in DMSO, were stored at -20°C until use at indicated final concentrations in Ussing chambers. Filters used for experiments to prevent distension had a pore size of 0.45 μM and a diameter of 2.5 cm (MF-Millipore, HABP02500, Merck Millipore Ltd., Ireland, Tullagreen, Carrigtwohill, County Cork, Ireland).

### Statistical analysis

Data are expressed as the median and [Q_25_/Q_75_] due to non-Gaussian distribution of most data. The amplitude of the secretory response was calculated as the difference of the I_sc_ (ΔI_sc_, μA cm^-2^) before and after serosal and mucosal pressure application, respectively. For pharmacology studies, distension induced secretory response was compared in a paired design before and after addition of the respective drug in the same tissue or in tissues from the same animal tested in different chambers (except from experiments with piroxicam in porcine tissue since control values were pooled from two sets of experiments). Secretory responses, in experiments with mucosal placed filters, were analyzed in a paired manner in tissue preparations from the same individual investigated in distinct chambers (human) and in an unpaired manner in porcine tissue preparations. Due to limited human tissue availability, secretory response data from the distinct regions were pooled for some of the statistical analysis (as indicated in the results section). In the case of Gaussian distribution (tested with the Shapiro-Wilk normality test), for analysis of data from paired experiments, paired Student’s t test or one-way repeated measures ANOVA followed by Holm Sidak’s multiple comparison test was used to test for significant differences. In case of not normally distributed data, the Wilcoxon signed rank test or Friedman test followed by Dunn’s multiple comparison test was applied. Parametric and non-parametric data from unpaired experiments were tested using the Student’s t test and the Mann-Whitney rank sum test or the ordinary one-way ANOVA and the Kruskal-Wallis test followed by Dunn’s multiple comparison test, respectively. Statistical analysis was performed using GraphPad Prism 9.0.0 (GraphPad Software Inc., La Jolla, CA, USA; https://www.graph pad.com). P < 0.05 was considered statistically significant. In the text and within figure legends, n numbers are given as numbers of tissue preparations.

## Results

### Secretory response to P_ser_ and P_muc_

**Fig 1:**
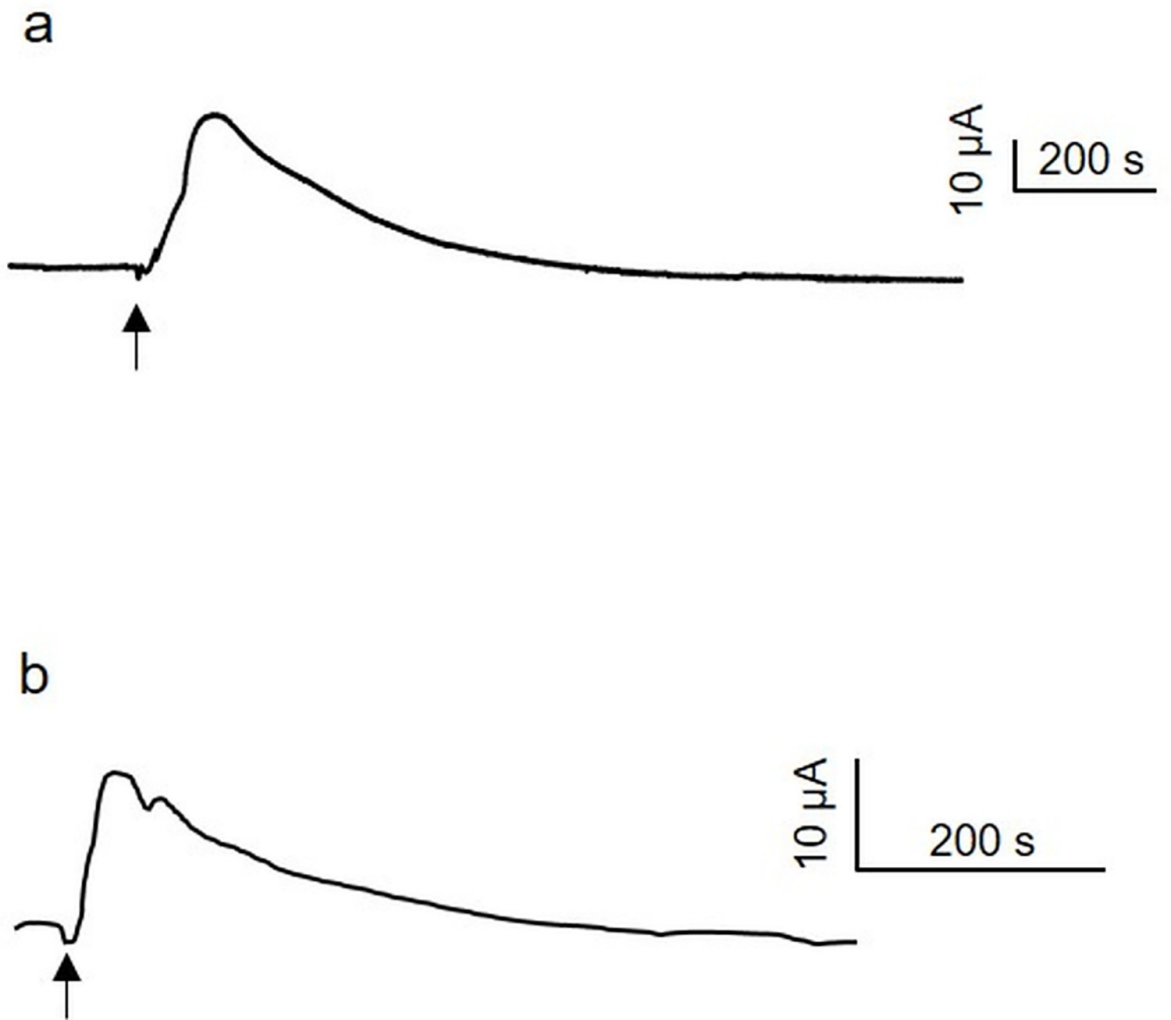
Traces of the secretory response to serosal pressure application (P_ser_). Representative short circuit current traces evoked by P_ser_ in (a) human sigmoid (20 mmHg) and (b) porcine transverse (60 mmHg) colonic tissue. Arrows indicate onset of P_ser_ lasting for 60 s.

Pressure application in the serosal or mucosal bath (P_ser_ or P_muc_, respectively) evoked a gradual increase in I_SC_ during pressure application and kept increasing even after pressure has been released. Fig 1 shows a representative trace of the secretory response to P_ser_ in human and porcine colonic tissue. The peak response occurred 105 [97/120] sec (n = 29), 110 [98/139] sec (n = 41), 102 [94/115] sec (n = 30) and 108 [97/126] sec (n = 93) after onset of P_ser_ (20 mmHg) in human ascending, transverse, descending and sigmoid colon, respectively; the corresponding values for P_muc_ were 143.5 [127/204] sec (n = 18), 147 [129/195] sec (n = 8), 139 [90/218] sec (n = 27) and 150 [74/185] sec (n = 27). In the porcine colon, the secretory response peaked after 48 [42/54] sec for P_ser_ and after 48 [44/60] sec for P_muc_, respectively (n = 68, each). The response was always monophasic and lasted 769 [654/899] sec (n = 40), 973 [750/1105] sec (n = 44), 585 [485/785] sec (n = 33) and 680 [513/884] sec (n = 99) in the human ascending, transverse, descending and sigmoid colon and 423 [363/500] sec in the porcine colon (n = 68), respectively, for P_ser_. The corresponding data for P_muc_ were 789 [633/1083] sec (n = 18), 707 [488/988] sec (n = 6), 538 [277/781] sec (n = 9), 624 [331/794] sec (n = 29) in the different human colonic segments and 360 [311/411] sec in the porcine colon (n = 68).

**Fig 2:**
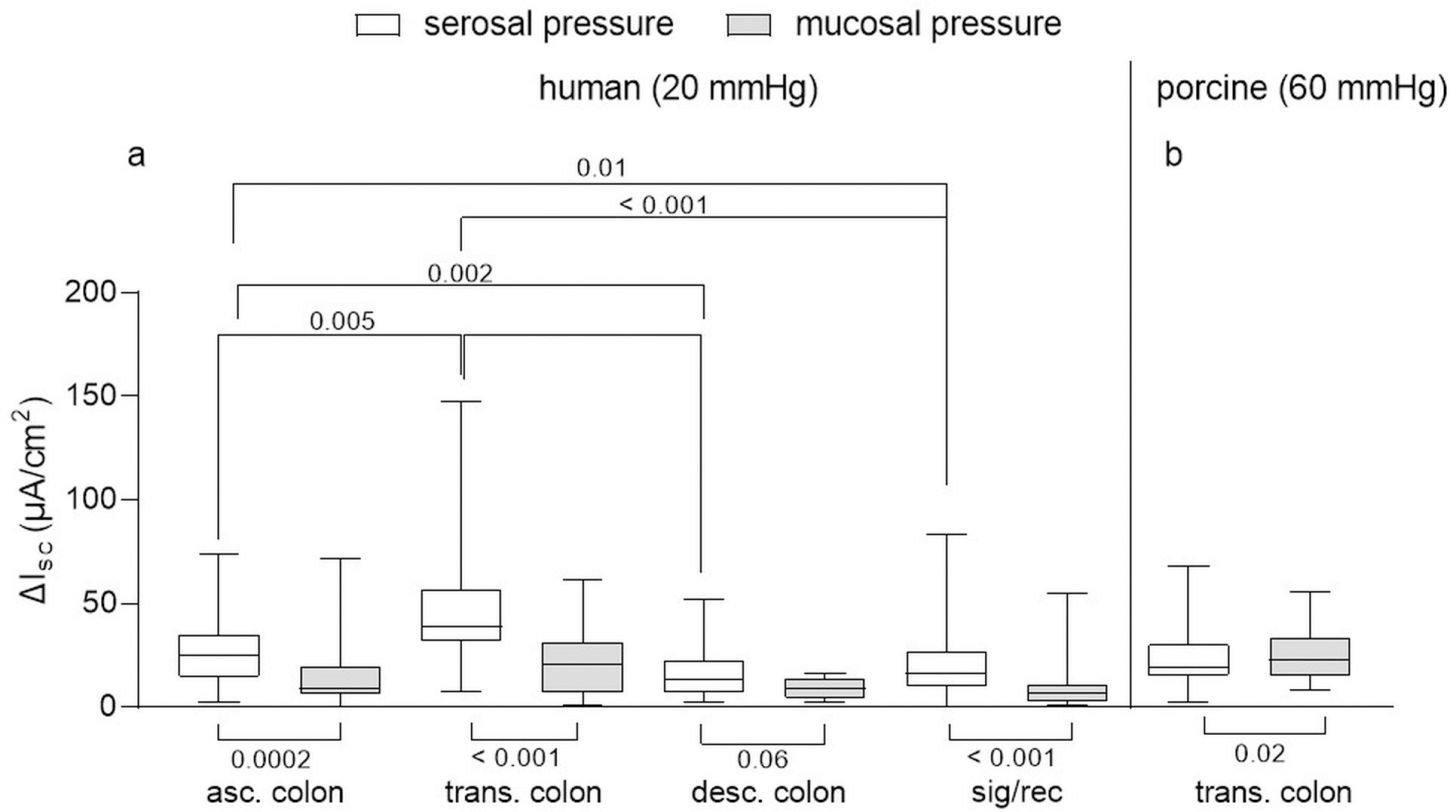
Secretory responses to serosal (P_ser_) and mucosal (P_muc_) pressure application. (a) human tissue: Responses to P_ser_ 20 mmHg were significantly greater than those P_muc_ application in all colonic regions investigated despite of the descending colon (bottom brackets; ascending colon = asc. colon, n = 51; transverse colon = trans. colon, n = 45; descending colon = desc. colon; n = 37, sigma–rectum regions = sig/rec, n = 109; Mann-Whitney test, numbers below brackets indicate p values). In the transverse colon the distension-induced secretory response was significantly greater than in all other regions (top brackets, Kruskal-Wallis (p < 0.001) test with Dunn’s multiple comparison test and p values above brackets). (b) porcine tissue: Responses to P_ser_ 60 mmHg were significantly smaller than those to P_muc_ in the transverse colon (n = 80); Wilcoxon signed rank test, p value below bracket). Data shown are the medians with the 25^th^ and 75^th^ quartiles as a box plot and the minima and maxima as a whisker plot.

In human tissues, P_ser_ evoked in all regions, except from the descending colon, a larger response than P_muc_ (Fig 2A). In contrast, in the porcine colon, P_muc_ evoked a larger response than P_ser_ (Fig 2B).

**Fig 3:**
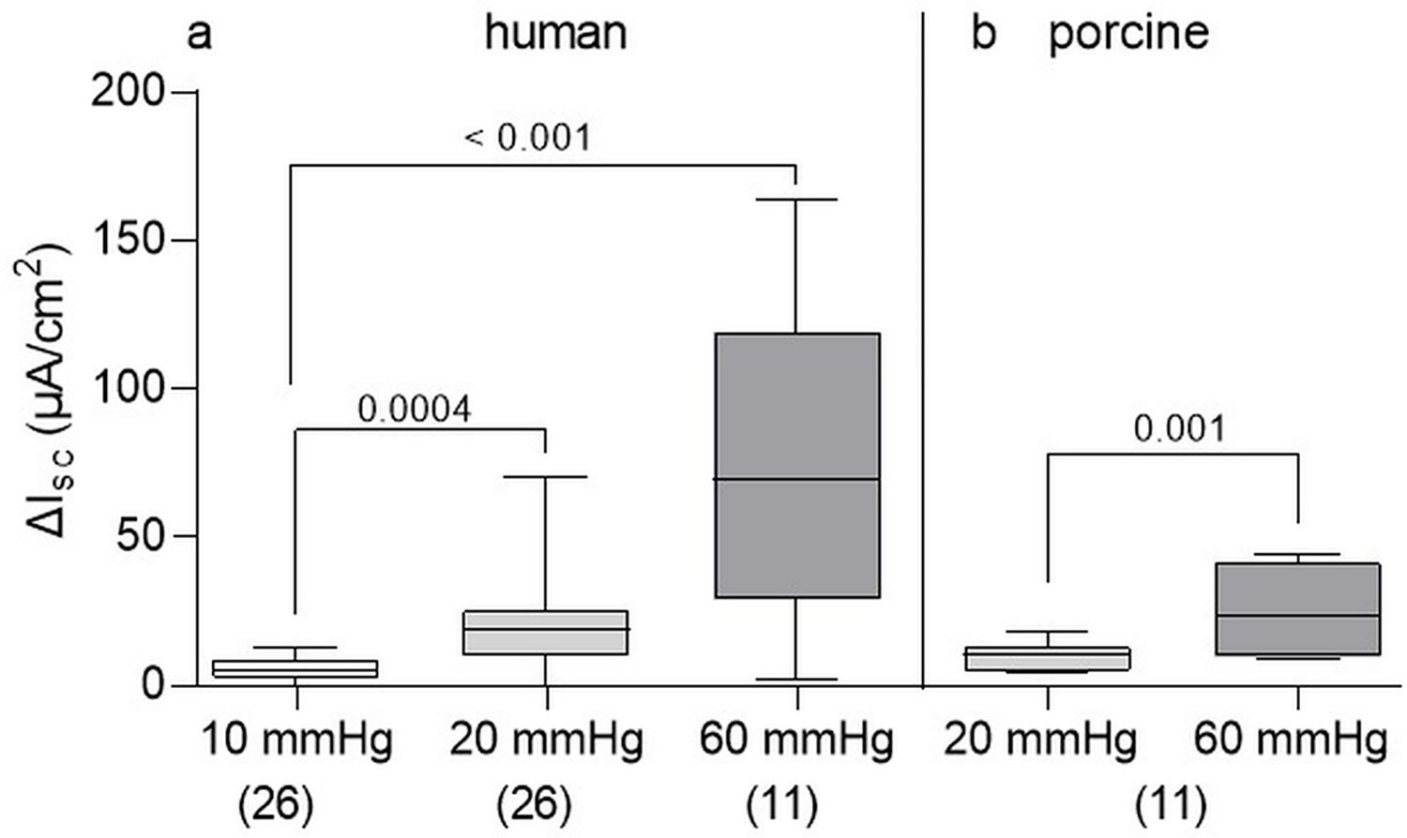
Secretory response to different pressure values. In human (a) and porcine (b) colonic tissue preparations, secretory responses to P_ser_ significantly increased with increasing stimulus strength. Human: Intestinal regions pooled; Kruskal-Wallis test (p < 0.001) with Dunn’s multiple comparison test and p values above brackets; porcine: Paired t test, p value above bracket. Data shown are the medians with the 25^th^ and 75^th^ quartiles as a box plot and the minima and maxima as a whisker plot. N numbers are given in parenthesis.

P_ser_ in human colon at pressures of 10 mmHg, 20 mmHg and 60 mmHg evoked increasing secretory responses of 4.9 [2.6/8.8] μA cm^-2^, 18.8 [10.0/25.5] μA cm^-2^ and 69.9 [29.1/119.2] μA cm^-2^, respectively. Both, secretory responses to the 20 and the 60 mmHg stimulus, were significantly greater than the response to the 10 mmHg stimulus (Fig 3A). In contrast, P_ser_ at 20 mmHg evoked a significantly smaller response in porcine colon compared to human colon (unpaired t test, p = 0.03). However, in porcine colon, the increase in I_sc_ induced by P_ser_ at 60 mmHg was significantly smaller than the secretory response evoked by the 60 mmHg in the human colon (23.3 [9.8/41.6] μA cm^-2^ vs. 69.9 [29.1/119.2] μA cm^-2^, unpaired t test, p = 0.008). Secretory responses in porcine colon evoked by P_ser_ at 60 mmHg were significantly greater than those evoked by P_ser_ at 20 mmHg (Fig 3B) and comparable to those evoked by 20 mmHg in human colon (p = 0.25).

In human colon, we initially studied P_ser_ induced secretion at various pressures in more detail. We found responses at pressures as low as 2 mmHg which gradually increased with larger pressure values. There was a highly significant correlation between pressure and secretory response (p < 0.001, Pearson r = 0.99). Likewise, there was a strong association between secretory responses at various pressures and the resulting distension volumes (p < 0.001, Pearson r = 0.99). We did not perform similar experiments after P_muc_ as we were afraid to damage epithelial cells at high pressure values. For further experiments in human tissue we used 20 mmHg pressure as this stimulus evoked reproducible and reliable responses. It also had the advantage that any pharmacology would reveal enhancing or reducing effects. Comparable to results in the human colon, we found a strong correlation between applied volumes to induce a 20 mmHg and a 60 mmHg pressure, respectively, and the degree of secretion in the porcine colon (p < 0.001, Spearman r = 0.80).

Interestingly, in porcine tissues, a median of 194.5 [160.5/412.5] μl was necessary to induce P_ser_ at 60 mmHg evoking a secretory response of 20.4 [9.9/34.5] μA cm^-2^. This was comparable to the P_ser_ evoked secretion in human colon at 20 mmHg: the secretory response was 18.7 [10.6/25.5] μA cm^-2^ at a volume distension of 290.0 [266.7/327.5] μl. Therefore, we used for all experiments in porcine colon a pressure value of 60 mmHg.

### Mechanically induced secretion was reproducible

**Fig 4:**
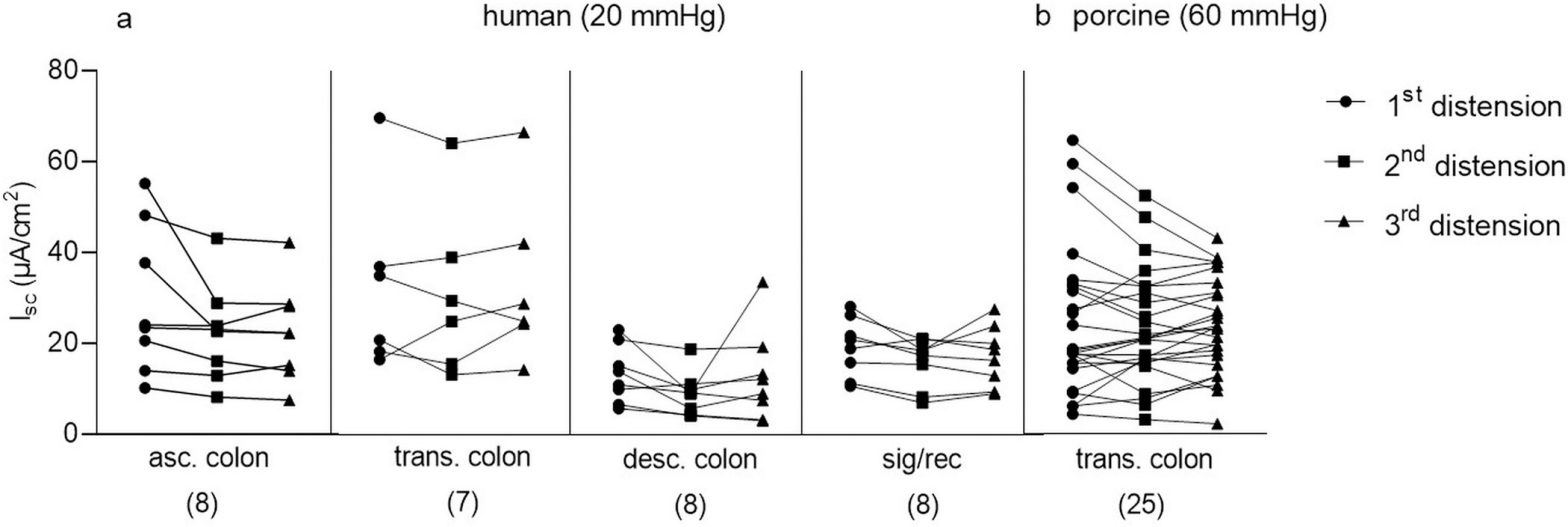
Secretory response to three times repeated P_ser_. In human (a) and porcine tissue (b), responses to three times repeated P_ser_ 20 mmHg (human) and 60 mmHg (porcine) were not significantly different from each other (human: Repeated measures ANOVA: n.s. with Holm-Sidak’s comparison test: n.s.; porcine: Friedman test: n.s. with Dunn‘s multiple comparison test: n.s.). Ascending colon = asc. colon; transverse colon = trans. colon; descending colon = desc. colon; sigma–rectum regions = sig/rec. Data shown are the individual values for the first, second and third pressure application, respectively. N numbers are given in parenthesis.

In both, human and porcine colonic tissue preparations, three consecutive P_ser_ (30 min in between) at 20 mmHg (human) and 60 mmHg (porcine) induced comparable secretory responses (Fig 4). The reproducibility was observed in the porcine and in all regions of the human colon. We only tested reproducibility after P_ser_ as all pharmacological interventions were performed after P_ser_.

### Distension evoked secretion depended on Cl^-^ and HCO_3_^-^ fluxes

**Fig 5:**
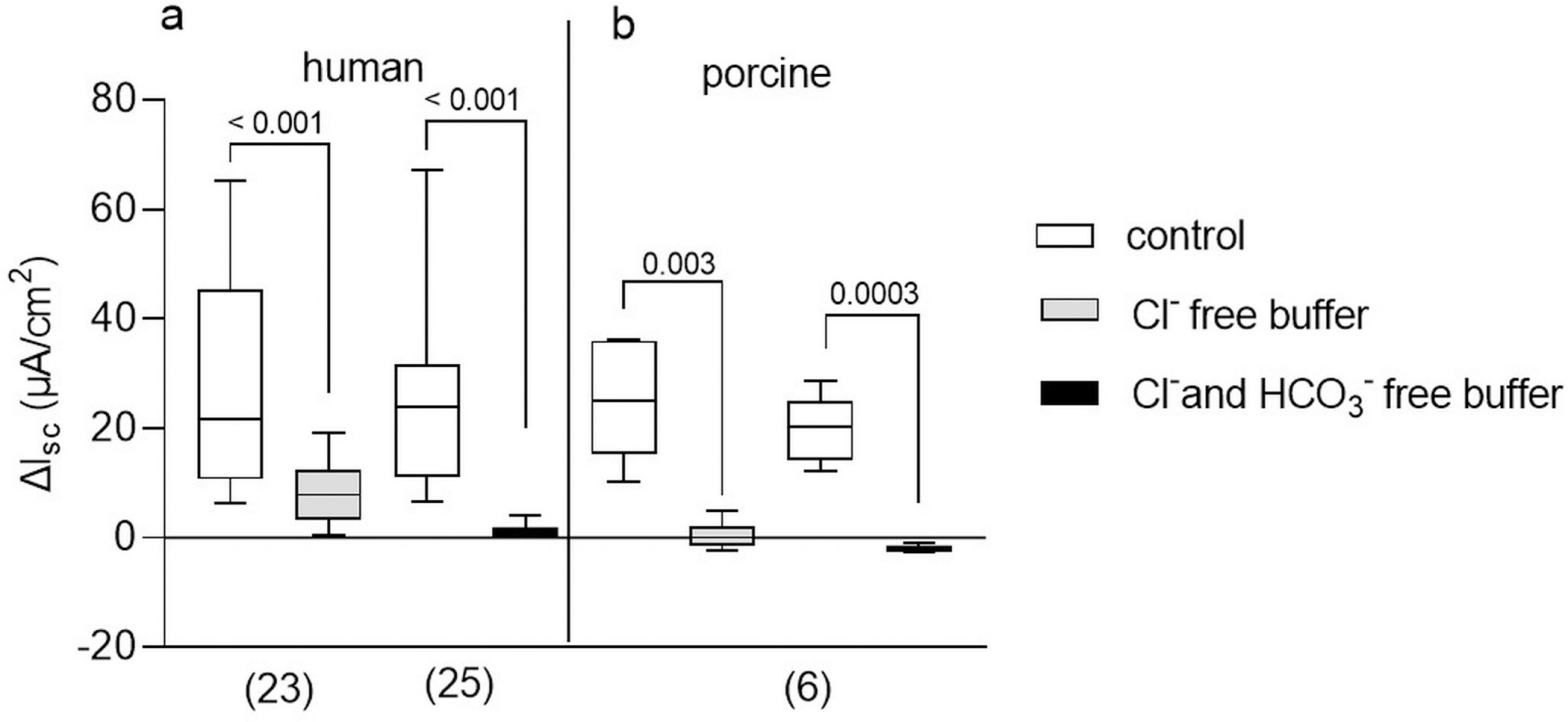
Ionic basis of the distension induced secretory response. Distension induced secretory responses in human (a) and porcine (b) colonic tissue evoked by P_ser_ was significantly reduced by depletion of chloride (Cl^-^) and Cl^-^ and bicarbonate (HCO_3_^-^), respectively (human: Wilcoxon test; porcine: Paired t test; p values above brackets). Data shown are the medians with the 25^th^ and 75^th^ quartiles as a box plot and the minima and maxima as a whisker plot. N numbers are given in parenthesis.

In both species, the distension-induced secretion was mainly a result of Cl^-^ secretion, with an additional strong HCO_3_^-^ component in human tissue (Fig 5A). On average, in Cl^-^ free buffer solution, the secretory response to P_ser_ at 20 mmHg was reduced by 71% in the human colon and 98% in porcine colon at 60 mmHg. Cl^-^ and HCO_3_^-^ depleted buffer solution caused a more pronounced reduction in the distension induced secretion in human colon while the response was not further reduced in porcine colon (Fig 5B).

### TTX sensitivity of distension evoked secretion

**Fig 6:**
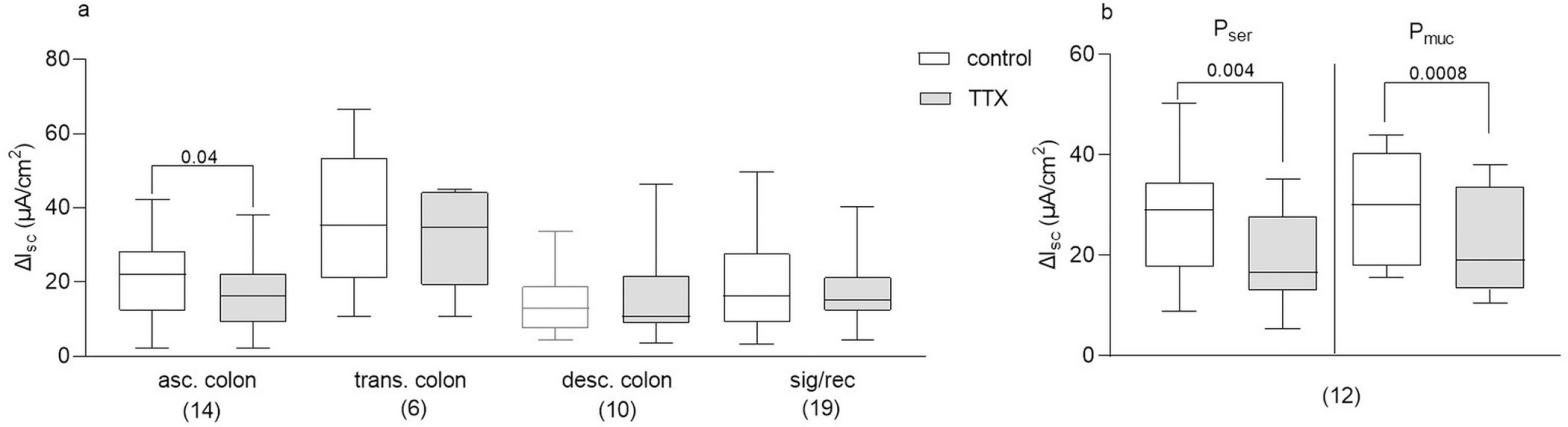
TTX sensitivity of the distension induced secretory response. Incubation of tissues with TTX prior to P_ser_ (and P_muc_ in porcine tissue) led to a reduced secretion only in the human ascending colon (a), and to a significant reduction in the secretory response in the porcine colon (b). Paired t test and Wilcoxon test; p values above brackets. Data shown are the medians with the 25^th^ and 75^th^ quartiles as a box plot and the minima and maxima as a whisker plot. N numbers are given in parenthesis.

In human transverse, descending and sigmoid colon TTX insignificantly reduced P_ser_ evoked secretion by 1.7%, 17.1% and 6.7%, respectively (Fig 6A). We found only in ascending colon a significant TTX sensitive component of about 27%. Secretion evoked by P_muc_ was TTX insensitive in all colonic regions (11.8 [5.3/17.3] μA cm^-2^ vs. [9.9/16.9] μA cm^-2^, p = 0.74, Wilcoxon test, pooled data).

In porcine colon, however, we did observe a significant reduction in distension evoked secretion by TTX of about 26% for P_ser_ and 27% for P_muc_ (Fig 6B).

### Prostaglandins were involved in the distension induced secretion

**Fig 7:**
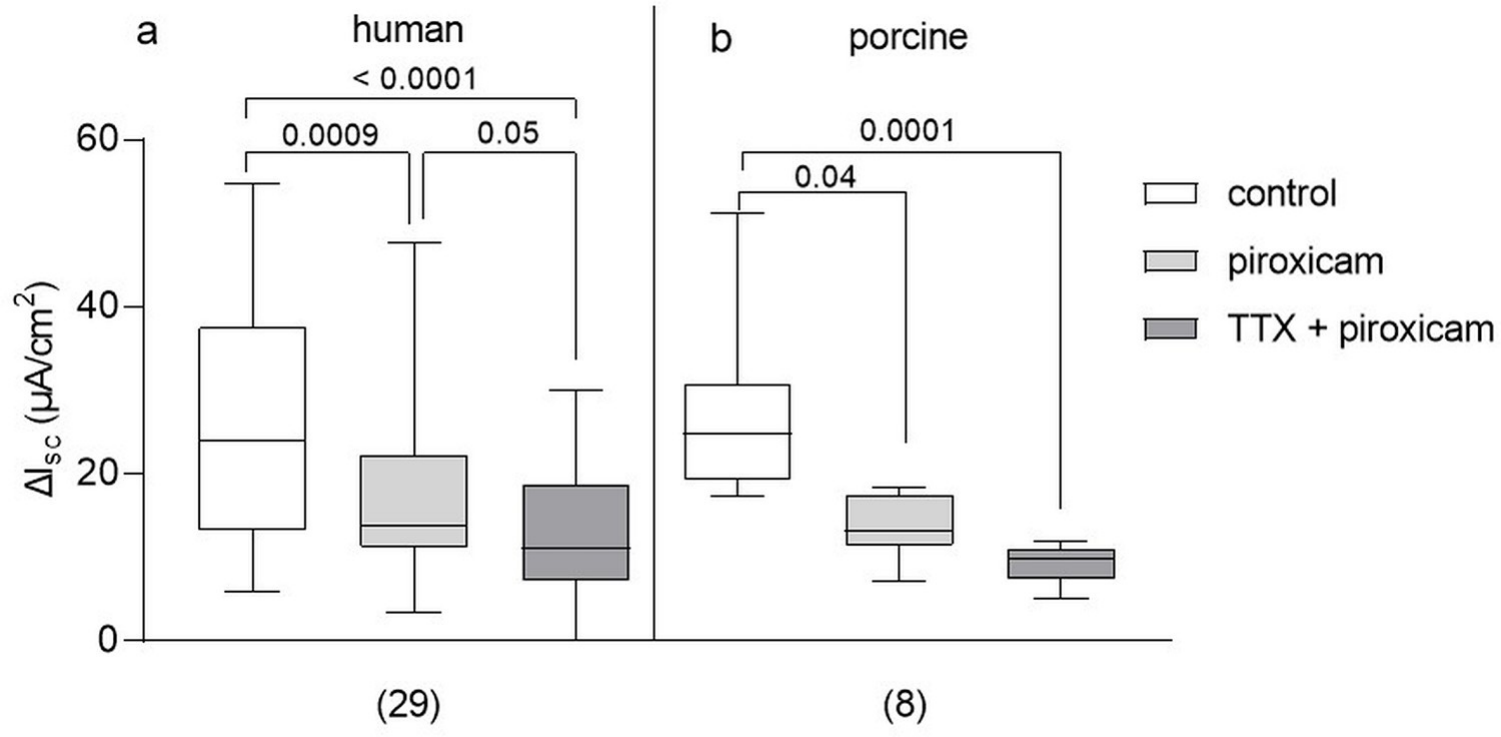
Involvement of prostaglandins in mediating the distension induced secretory response. Addition of piroxicam prior to P_ser_ reduced secretory response in human (a) and porcine tissue (b). Simultaneous incubation with TTX further reduced the response in human tissue (human: Friedman test with Dunn’s multiple comparison test; porcine: Kruskal-Wallis test with Dunn’s multiple comparison test; p values above brackets). Data shown are the medians with the 25^th^ and 75^th^ quartiles as a box plot and the minima and maxima as a whisker plot. N numbers are given in parenthesis.

Preventing PG synthesis by addition of piroxicam significantly reduced P_ser_ evoked secretion in both, human and porcine colon (Fig 7). The PG mediated response amounted to 42% and 52% in human and porcine colon, respectively.

In human colon, piroxicam unmasked a small but significant TTX sensitive response (Fig 7A) with a reduction by 18.6%. In porcine colon, the TTX sensitive component remained as the reduction was 49.4% in piroxicam treated tissues but further increased to 66.4% after additional incubation with TTX (Fig 7B). This suggested that the TTX sensitive component in porcine colon was unrelated to PG release and that the PG release itself was nerve independent in both species. This was supported by findings that TTX had no effect on PGE_2_ induced secretion (human: 85.5 [64.4/121.7] μA cm^-2^ vs. 87.6 [47.1/107.6] μA cm^-2^, n = 14, Wilcoxon test, p = 0.86; porcine: 22.3 [15.6/29.6] μA cm^-2^ vs. 29.0 [22.2/34.9] μA cm^-2^, n = 11, unpaired t test, p = 0.22).

### Distension evoked secretion involved mechanosensitive synapses in human colon

The surprisingly low TTX sensitivity to P_ser_ evoked secretion in human transverse, descending and sigmoid colon, prompted us to test whether mechanical stimulation may cause increased synaptic release without involvement of TTX sensitive soma spikes. We used ω-conotoxin GVIA as a potent blocker of synaptic activation of enteric neurons [29–31] and hence as a tool to identify mechanically induced synaptic release. P_ser_ induced secretion in human colonic tissue was significantly smaller (15% reduction) in the presence of ω-conotoxin GVIA (15.3 [24.6/11.4] μA cm^-2^ vs. 13.1 [7.7/15.5] μA cm^-2^; n = 12, paired t-test, p = 0.02).

The above finding suggested release of neurotransmitter, very likely at the neuro epithelial junction. This is supported by the following results: serosal application of the cholinesterase inhibitor neostigmine (1 μM) enhanced the P_ser_ induced secretion (15.4 [5.2/40.2] μA cm^-2^ vs. 21.9 [8.4/40.3] μA cm^-2^ (n = 19, Friedman test followed by Dunn’s multiple comparison test, p = 0.04), which was not affected by addition of TTX (23.4 [7.3/26.0] μA cm^-2^).

### Tension forces are the appropriate stimulus to induce secretion in human and porcine colonic tissue

**Fig 8:**
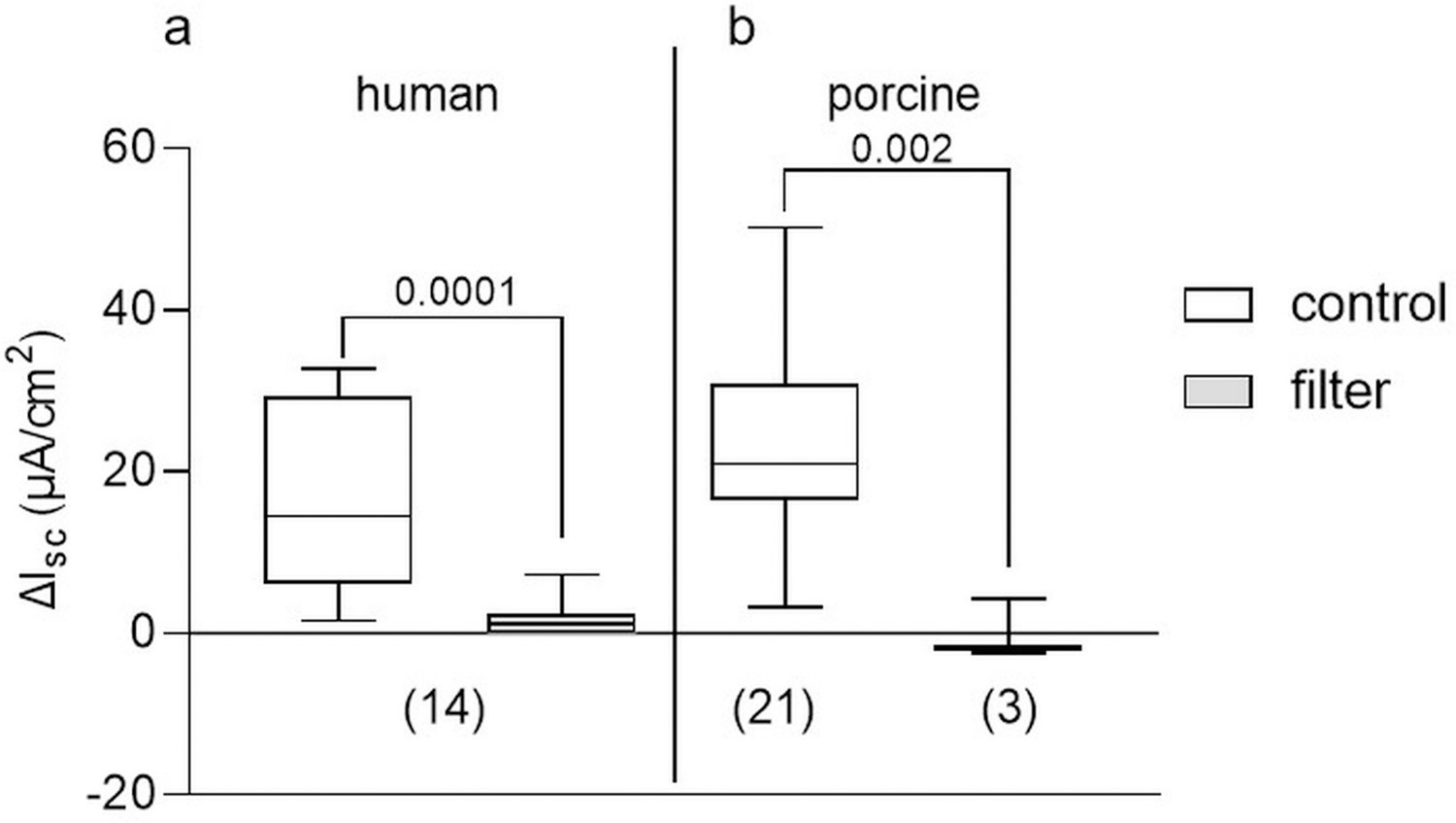
Secretory response to tensile forces. Prevention of tissue distension during P_ser_ by a mucosal placed filter significantly reduced the secretory response in human ((a); Wilcoxon test) and porcine (b) colonic tissue (unpaired t test); p values above brackets. Data shown are the medians with the 25^th^ and 75^th^ quartiles as a box plot and the minima and maxima as a whisker plot. N numbers are given in parenthesis.

Distension of the tissue by P_ser_ was greatly impaired by placing a filter on the mucosal side in both, human and porcine tissues (see S1 and S2 Videos). The restriction of tissue distension reduced the secretory response by 89% in human and 100% in porcine tissue, respectively, and hence was significantly lower compared to values recorded in control tissues (Fig 8). Therefore, tension forces rather than compression forces evoke secretion in human and porcine colonic tissue.

## Discussion

In the current study, we investigated epithelial responses of human and porcine colonic tissue to mucosal and serosal distension to evaluate similarities and differences between these two species in order to assess species comparability.

In both species, distension of the intestinal wall reproducibly evoked a secretory response, as it has already been shown for many other species in small and large intestinal regions [7–11,15]. In our study, the secretory response peaked at 1 to 2 min after onset of P_ser_ and lasted for 6 to 16 min in porcine and human colonic tissue, respectively. This is a quite long time laps between the applied distension stimulus and the epithelial secretory response when compared to the promptly initiated peristaltic reflex upon wall distension of the human colon [32]. However, our results are in accordance to values e.g. from the rat distal and rectal colon, with peaks of distension induced secretion 1 to approximately 7 min after pressure application and duration of the secretory responses between 10 and 30 min [8,9,11,15]. It is also in accordance with results from *in vivo* studies showing the close (temporal) association between increased intraluminal pressures representing enhanced muscle contractions followed by PD changes indicating electrolyte movement [12–14]. Based on this finding, the distension stimulus used in our *in vitro* setup seems to mimic physiological responses observed in *in vivo* studies quite well [33,34]. Moreover, the slower response fits to activation of a mostly non-neuronal component.

The underlying reason for an increased and ongoing secretion in response to a higher intestinal pressure in the colon, might be a mucosa protective, pro-propulsive and anti-ileus mechanism: during the passage through the colon, water is absorbed from the intestinal contents and hence stool viscosity increases [35]. It is plausible that a smooth transport of larger amounts requires a more liquid intraluminal content. This is also supported by the study by Itasaka and colleagues, confirming that in the rat colon, fecal pellets with a physiological diameter would be able to induce secretion [11]. Distension evoked secretion, however, was not only observed after mucosal pressure, which was applied to mimic a widening of the intestinal lumen as it would occur when the circular muscle layer relaxes, but also after serosal pressure application; the latter would represent rather a decreased intestinal lumen as it occurs during (circular) smooth muscle contraction. Moreover, we observed in the human colon a larger response to P_ser_ than to P_muc_. This suggests that also a constriction of the colon induced lubrication in order to prevent damage during enhanced contractile activity by advantageously affecting content viscosity and preventing obstruction and ileus [36]. However, in the present study, in contrast to the human colon, in the porcine colon P_muc_ induced a greater secretory response than P_ser_ and a greater P_ser_ was needed to induce secretory responses comparable to those in the human colon. It has been shown that the mechanical strength of colonic tissue is mainly determined by the submucosa and muscular layers whereas serosa and mucosa have no significant stiffness [37,38]. Hence, naturally existing different amounts of these layers in different species and slightly different composition of our human and porcine mucosa/submucosa preparations might have influenced the responses to distension from the serosal and mucosal side in human and porcine tissue, respectively. In porcine tissue, P_ser_ at 60 mmHg induced secretory responses comparable to those in human tissues at P_ser_ 20 mmHg. We have no plausible explanation for the higher pressures needed in porcine compared to human colon to induce comparable secretory responses. However, since we found a positive correlation between injected volumes and secretory responses and reproducible responses to the chosen pressures in both species, we think, that results obtained are reliably.

In both the human and porcine colon, the secretory response after P_ser_ was mainly Cl^-^ driven. However, in the human colon HCO_3_^-^ also seems to play a distinct role. This agrees with findings that the EFS induced secretory response in the human colon depended on Cl^-^ and HCO_3_^-^ fluxes [6] but was based on Cl^-^ secretion in the distal colon of piglets [23]. Contribution of both, Cl^-^ and HCO_3_^-^ seems to be a species specific characteristic of the distension induced secretory response of the human colon, since secretion was also almost entirely Cl^-^ driven in guinea pigs and rats [10,15–17]. While to our knowledge no experiments on distension induced secretion were conducted in mice, carbachol stimulated secretion in murine colonic tissue was also mainly based on Cl^-^ and only to a very small extent on HCO_3_^-^ [39,40].

There are basically three transduction mechanisms that may link mucosal deformation to ion secretion. First, activation of enteric circuits with mechanosensory neurons detecting deformation and initiating the secretomotor reflex. Second, direct activation of epithelial cells via mechanosensitive channels. Third, activation of other lamina propria cells such as immune cells releasing non-neuronal transmitters activating epithelial secretion. One main result of our study is the relatively small nerve-dependent component of the distension induced secretion. This is in strong contrast to the strong TTX sensitivity of intestinal motor activity upon intestinal wall distention, best known as the peristaltic reflex [41,42]. In the human colon, the TTX sensitive component of the distention induced secretory response was clearly masked by the effect of PGs. However, since ω-conotoxin GVIA reduced the response to P_ser_, involvement of mechanosensitive synapses in the transduction of the distension stimulus was clearly indicated. Indeed, probing neurites in cultured human enteric neurons caused spike discharge [43]. Alternatively, ω-conotoxin GVIA may block some epithelial calcium channels involved in Cl^-^ and HCO_3_^-^ fluxes, although the existence of such channels on epithelial cells has not been reported. Considering our results obtained in experiments with neostigmine, acetylcholine is very likely involved in transmitting activation of mechanosensitive synapses to target epithelial cells. Due to limited tissue supply, we were not able to test whether atropine reduced the neostigmine enhanced secretion, which would have strengthened this assumption. However, this idea is strongly suggested by the finding by Krueger and colleagues, who showed that neostigmine unmasked an atropine sensitive component of nerve evoked secretion [6]. Compared to our results, the TTX sensitive component of distension induced secretion in the colon of rodent species was greater, ranging from 30 to 90% [8–11,16,17]. Hence, with regard to this feature of distension induced colonic secretion, the porcine colon might model the human better than the traditionally used rodent species. However, the methods to induce serosal distension, as well as distension protocols in the cited studies varied to a great extent compared to ours (e.g. introduction of a hydrostatic gradient vs. reduction and re-injection of liquid volume vs. use of syringe pump injection, single and consecutive distension, duration of distension) which might also explain variances in the results obtained. Although speculative, a strong direct activation of the epithelium in response to mechanical deformation might be an advantage as dependency on nerves bare the risk that nerve damage prevents crucial lubrication. The recorded regional differences regarding the nerve mediated component of the response within the human colon might somehow reflect the greater need for direct, nerve-independent secretion upon distension in the more distal parts of the colon due to increasing stool viscosity and probably increased susceptibility for stasis. Whether the small nerve component is related to the size of the species can only be answered if more studies on larger animal species have been conducted.

In both species, we found a dominant PG mediated component of the distension induced secretory response which is in accordance to earlier studies in the rat colon [9,11,17], but in contrast to studies in the guinea pig colon [10]. The PGs may be released by a number of non-neuronal cells including epithelial cells, muscularis mucosae and immune cells all possessing mechanosensitive channels such as Piezo channels to sense and transduce mechanical stimuli [44–46]. In addition, COX-1 and even COX-2 is constitutively expressed in human and murine enteric neurons [44,47]. Since in both, human and porcine tissues, the piroxicam effect was still significant after neuronal blockade with TTX, PGs must directly act on epithelial cells, as also shown for the rat distal colon [9,11]. It has been shown that mechanosensitive channels in enterocytes activated a basolateral potassium conductance [48]. Likewise the cystic fibrosis transmembrane conductance regulator (CFTR) channel has been shown to be mechanosensitive and activated by stretch/tension forces [49]. This fits to our finding that the appropriate secretion inducing stimulus were tensile rather than compressive forces in both, human and pig colon. Hence, the TTX insensitive response in the human colon was likely a result of such a direct activation of CFTR channels. In addition, submucosal neurons in the pig and to a lesser extent in the human colon responded to stretch [28]. With regard to the dominant role we found for PGs for the distension-induced epithelial secretion in both, the human and the pig, it could be speculated that the known increased risk for lower intestinal haemorrhage or perforation [50] and exacerbation of colitis [51] upon the use of COX inhibitors/non-steroidal therapeutics, represents a potential underlying reason.

In summary, we could show that in human and porcine tissue, serosal as well as mucosal distention evokes a secretory response, which is stimulus-strength-dependent and mainly Cl^-^ driven with an additional significant HCO_3_^-^ component in the human colon. In both species, the contribution of nerves was rather small with a dominant role of PGs released from non-neuronal cells. The distension evoked response was primarily due to tensile forces.

From the current study the following conclusions can be drawn: (1) distension of the human and porcine colonic epithelium, mimicking relaxation or contraction, induces secretion; (2) distension induced secretion is mainly dependent on PG release with a rather small neuronal contribution; (3) certain features of the distension induced secretory response are species-specific. Based on this, the porcine intestine models the human one as good (or bad) as any rodent model.

## Supporting information

S1 Fig A porcine intestinal tract (with the stomach and small intestines removed) from a mature hybrid pig after removal from the carcass. Porcine tissue samples for Ussing chamber experiments were taken from the transverse colon approximately 10 cm distal from the last spiral gyri centrifugales of the spiral colon.
(TIF)

S1 Video Distension of human colonic tissue by serosal pressure application. Pressure application (20 mmHg) from the serosal side led to a visible distension of human colonic mucosa/submucosa preparations into the mucosal compartment of the Ussing chamber.
(MP4)

S2 Video Prevention of pressure-induced distension of human colonic tissue by a mucosal placed filter. A mucosal placed filter prevented distension of human colonic mucosa/submucosa preparations induced by a 20 mmHg pressure applied from the serosal side.
(MP4)
