# Supplementary figures and images for: Distension evoked mucosal secretion in human and porcine colon in vitro
Source: PLoS One. 2023 Apr 13;18(4):e0282732. doi: 10.1371/journal.pone.0282732 (PMC10101454; doi:10.1371/journal.pone.0282732)

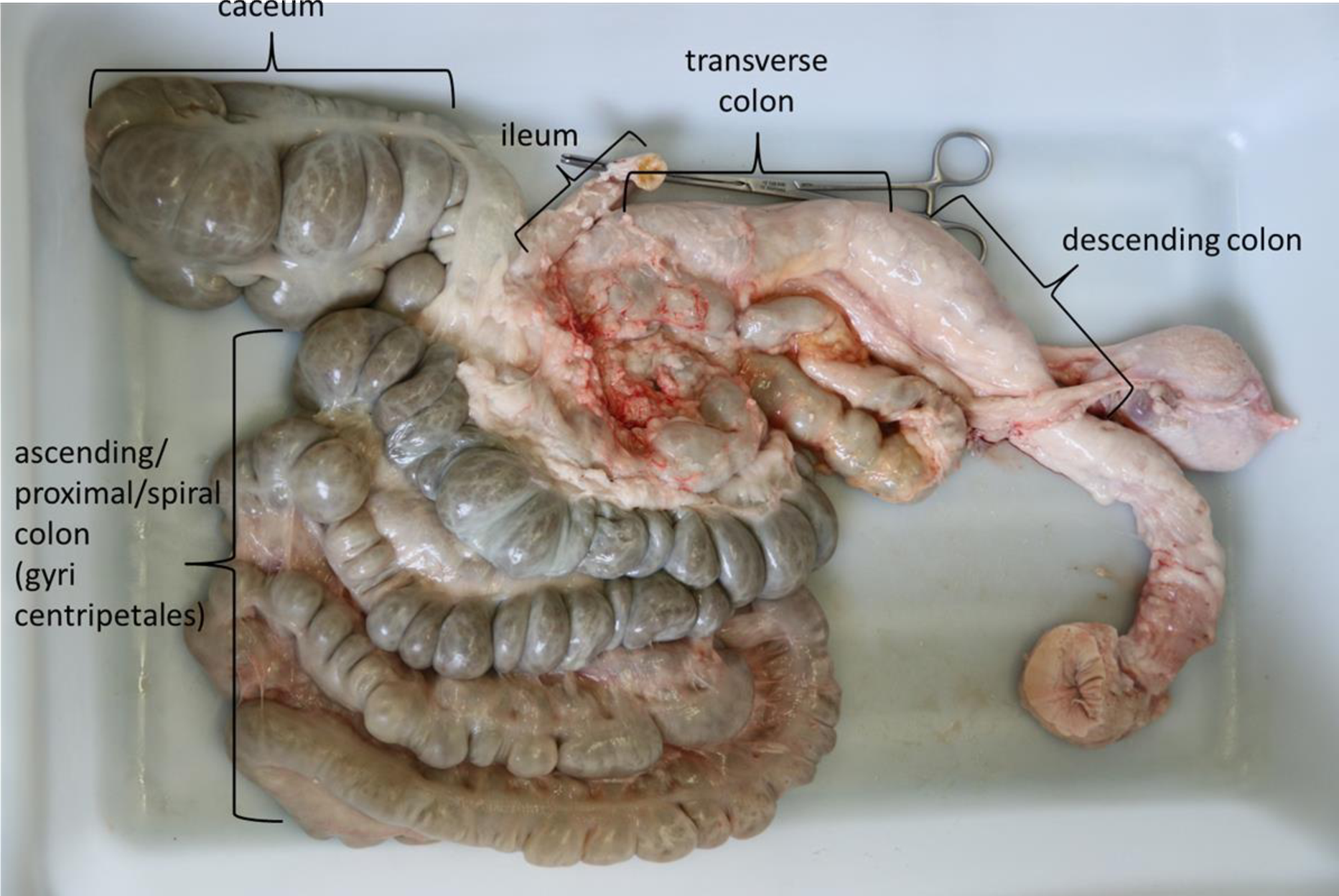

Supplement: S1 Fig — Porcine tissue samples for Ussing chamber experiments were taken from the transverse colon approximately 10 cm distal from the last spiral gyri centrifugales of the spiral colon. (TIF) [file pone.0282732.s001.tif]
